# Adolescents’ knowledge of HPV and sexually transmitted infections at public high schools in São Paulo: A cross-sectional study

**DOI:** 10.1016/j.clinsp.2022.100138

**Published:** 2022-11-17

**Authors:** Jose Maria Soares Junior, Hervillin Maria Creusa de Oliveira, Camilla Maganhin Luquetti, Lea Tami Suzuki Zuchelo, Eduardo Carvalho de Arruda Veiga, Juliana Zangirolami Raimundo, Francisco Winter dos Santos Figueiredo, Mayara Souza Alves, Isabel Cristina Esposito Sorpreso, Edmund Chada Baracat

**Affiliations:** a Disciplina de Ginecologia, Departamento de Obstetrícia e Ginecologia, Hospital das Clínicas, Faculdade de Medicina da Universidade de São Paulo, São Paulo, SP, Brazil; b Laboratório de Epidemiologia e Estatística, Centro Universitário do ABC, Santo André, SP, Brazil

**Keywords:** Adolescent health, Papillomaviridae, Vaccination, Knowledge, Health education, Public health

## Abstract

**Purpose:**

To assess the knowledge of students from public high schools in poor communities about HPV and Sexually Transmitted Infections and their attitude towards and prevention of such diseases.

**Patients and methods:**

Cross-sectional study with adolescents from public schools of São Paulo – Brazil. Participants were selected for an interview by a randomization program. A questionnaire about knowledge, attitudes, and preventive practices regarding STIs, including HPV, according to sex was administered and answers were analyzed by the Poisson regression model with robust variance.

**Results:**

Median age of the 269 participants was 16 years. The majority was of African descent (68.8%, n = 185), most (74%, n = 199) were religious and the vast majority (90.7%, n = 244) lived with their parents. The Poisson regression revealed statistically significant sex-related differences regarding the following questions: “Do you know how it is prevented?” (PR = 1.12 [1.03‒1.23], p = 0.007); “Have you ever been concerned with HPV?” (PR = 1.10 [1.02‒1.19], p = 0.011); “Have you ever sought health care due to concerns about HPV?” (PR = 1.09 [1.04‒1.14], p < 0.001); “Do you know what a Pap Smear is?” (PR = 1.24 [1.13‒1.36], p < 0.001); “Do you know what the cervix is?” (PR = 1.23 [1.13‒1.34], p < 0.001); “Do you know what cervical cancer is?” (PR = 1.13 [1.04‒1.22], p = 0.004).

**Conclusions:**

The present results show that adolescents from public schools in poor communities in São Paulo City know little about HPV and cervical cancer. Male adolescents know less than female adolescents and are less concerned with health care.

## Highlights

• Adolescents’ knowledge of HPV.
• Adolescents’ knowledge of sexually transmitted diseases.
• Female adolescents’ knowledge of HPV and sexually transmitted diseases.
• Male adolescents’ knowledge of HPV and sexually transmitted diseases.

## Introduction

Adolescence is the period when sexual life might initiate. Currently, most adolescents engage in sexual intercourse at an increasingly early age.^1^ Lack of knowledge and early engagement in sex render adolescents vulnerable to Human Papillomavirus (HPV) and other Sexually Transmitted Infections (STIs).

The prevalence of HPV in Brazil is 40% to 60%, and in the state of São Paulo, it is over 56%.^2^ The quadrivalent HPV vaccine was introduced in Brazil at no cost to the population in 2014, reaching a vaccination coverage of 100% of the target population; however, after changes in the vaccination strategy, the coverage dropped to 44.64% in 2015.^3^

Distribution of male condoms and HPV vaccination are strategies to reduce transmission of HPV and other STIs and prevent precursor lesions of cervical cancer among teenagers. Adherence to condom use and to vaccination are behaviors dependent on one's knowledge of these infections, and they may be influenced by sex, socioeconomic level, and educational, cultural, and religious backgrounds.^1^^,^^2^

A school is a place of learning for adolescents. It not only plays an important role in education, but it is also a setting for the promotion of reproductive and sexual health among youth.^4^^,^^5^

HPV infection is associated with age, for it is young people who are most prone to taking risks. In Brazil, the quadrivalent HPV vaccine is freely distributed to girls from 9 to 14 years old, boys from 11 to 14 years old, and immunosuppressed men and women, aged 9 to 45 years, living with HIV/AIDS, transplanted solid organs or bone marrow, and cancer patients.^6^^,^^7^

However, the authors don't know if public high schools in underprivileged areas are being able to transmit necessary information about HPV and STIs and to develop the interest of adolescents from low-income families in their sexual health.^1^^,^^2^ Therefore, the study's objective was to assess the knowledge of students from public high schools in poor communities in the city of São Paulo about HPV and STIs and their attitude towards such diseases, and the measures they take to prevent them.

## Methods

### Type of study, setting, and time span

This cross-sectional study assessing students from three public high schools was conducted by the Discipline of Gynecology of the Universidade de São Paulo (USP) between 2018 and 2019. The project was approved by the Ethics Committee of the “Faculdade de Medicina da Universidade de São Paulo” (FMUSP). The protocol number is 38719314.2.00000068.

**Fig. 1:**
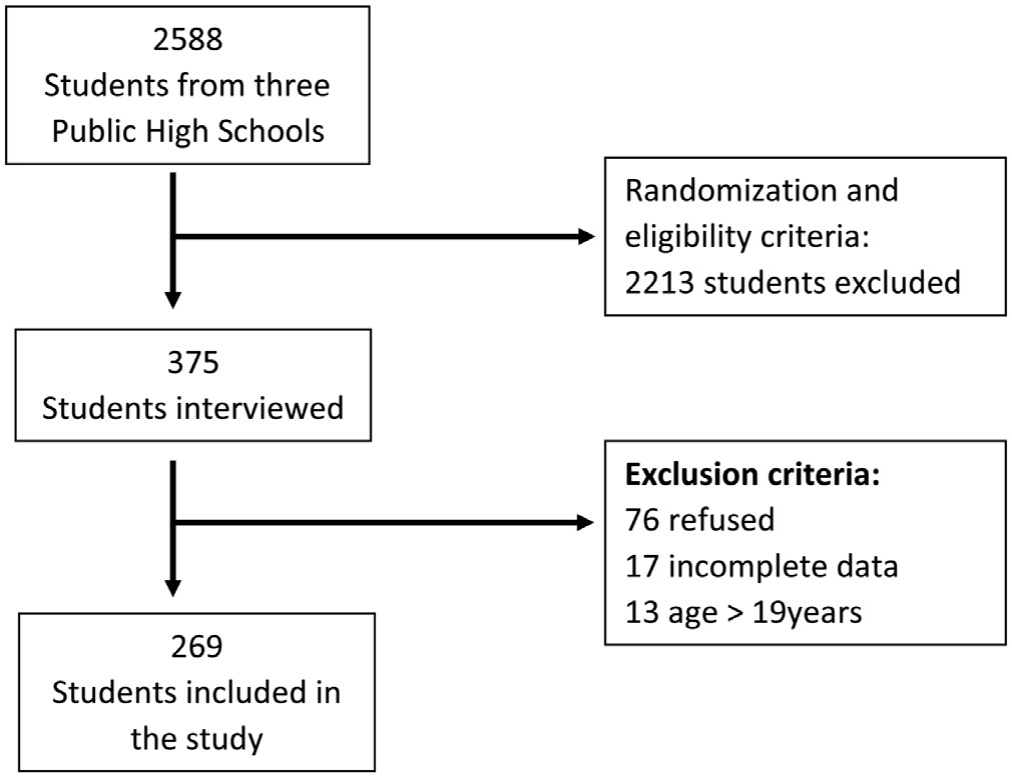
Flowchart of student inclusion.

Of the 2588 students from three state high schools located in the city of São Paulo, 375 were selected for an interview according to the following inclusion criteria: proper enrollment in a public school and 19 years of age or less. Fig. 1 shows the flowchart of student inclusion.

### Sample size

The sample size was estimated at 253 students, given a confidence level of 85% and power test of 80% in line with the Kops et al. (2019)^8^ study, which evaluated adolescents’ level of HPV knowledge.

### Randomization

The students were selected for an interview by a computer-generated randomization list. If a student on the list was unwilling to participate, no substitution was made (the refusal rate was 20%). Prior to data collection, all interviewers were trained and certified.

### Data collection and instrument used

The questionnaire was designed by the authors in accordance with the scientific literature. It addresses the student's knowledge of the prevention, transmission, and consequences of STIs, including HPV, as well as their attitude toward the diseases and their preventive practices. The questionnaire contains 13 items grouped into four categories: knowledge, attitudes, health practices, and HPV vaccination.

The questionnaires were administered by students from the *Programa Institucional de Bolsas de Iniciação Científica* (PIBIC) (Institutional Scientific Initiation Scholarship Program), which is a program of prescientific initiation and technology innovation at USP. Before the questionnaires were handed out, the participants were informed about the research objectives, and they signed an informed consent form. No student identification was required on the questionnaire. After the students completed the questionnaires, any questions they had were answered for further clarification.

A pilot study was conducted in 2018 (n = 50) to evaluate and improve the questions and ensure comprehension. Validation of the questionnaire was based on experts’ analyses (five researchers with substantial experience in field research and epidemiological studies), semantic analyses, and pretesting. After completion, each questionnaire was analyzed and then tabulated. Thirty pilot interviews were recorded, and three interviewers listened to each interview. The questionnaire was then improved further, and the interviewers received instructions for administering an interview and training as well as shown in Supplementary Material.

In addition to the questionnaire above, a sociodemographic questionnaire was drawn up with questions about birthplace, age, ethnicity, sex, religion, family income, the social welfare program (*Bolsa Família*), sexual intercourse, and family nucleus.

### Questionnaire validation

The questionnaire was validated using a factor analysis of answers and questions, following the methods of factorial analysis. Intraobserver and interobserver reliability was tested using the κ method, and consistency was measured with Cronbach's alpha coefficient.

### Statistical analysis

The data were tabulated and analyzed by the Stata® 14.2 (Stata Corp, College Station, USA) software. Answers to questions 1, 2, 3, 6, 7, 8, 9 10, 11, 12, and 13 were dichotomized into “presence of knowledge” when the answer was “I know a little” or “I know”, and “lack of knowledge”, when the answer was “I don't know”, “I heard of” or “I know partially”.

On the other hand, answers to questions 4 and 5 were dichotomized into “I was worried/I sought health care”, “when the answer was “frequently”, “I'm aware”, or “I worried a lot”, and into ‘I wasn't worried/I didn't seek health care’ when the answer was “never” or “sometimes”.

The dataset^9^ is published in Harvard data verse: https://doi.org/10.7910/DVN/OCNLUF.

Participants were divided into 2 groups according to their sex: male or female. Qualitative variables were reported as absolute and relative frequencies, and the quantitative variable ‘age’ was expressed by the median and confidence interval. The Chi-Square and the Mann-Whitney tests were used to analyze homogeneity between the two groups in relation to socioeconomic variables.

The Poisson regression model with robust variance was used to analyze the answers to questions about students’ knowledge, attitudes, and preventive practices with respect to STIs, including HPV, according to sex. The level of statistical significance for the sample was set at 5%. The model was adjusted according to income, age, ethnicity, grade repetition, cohabitation with parents, and religion.

## Results

The internal consistency of the questionnaire, expressed by Cronbach's alpha coefficient, 0.78 in this case, was substantial.

The total study population consisted of 269 high school students in which themedian age was 16 years (95% CI 15.9‒16.1; p = 0.310). The majority (74.7%, n = 201) lived in the city of São Paulo, and most of these (59.1%, n = 156) resided in neighborhoods near their schools. A considerable number of the students (68.8%, n = 185) were of African descent.

Over half of the adolescents (50.6%, n = 136) declared they had already engaged in sexual intercourse; most (74%, n = 199) had religious beliefs; a vast majority (90.7%, n = 244) lived with their parents; and over two-thirds (67.3%, n = 181) had never failed a grade in school. Despite the fact that most (60.6%, n = 163) received less than the minimum salary, a very large number (80.8%, n = 215) were not enrolled in the country's social welfare program, the so-called *Bolsa Família* (Table 1).

**Table 1:** Socioeconomic variables of public high school students according to sex.

Characteristics	Female	Male	Total	p^a^
	n (%)	n (%)	n (%)	
City				
In São Paulo	91 (74.6)	110 (74.8)	201 (74.7)	0.964
Out of São Paulo	31 (25.4)	37 (25.2)	68 (25.3)	
Neighborhood				
Near school	72 (61.0)	84 (57.5)	156 (59.1)	0.567
Far from school	46 (39.0)	62 (42.5)	108 (40.9)	
Ethnicity				
White	38 (31.1)	46 (31.3)	84 (31.2)	0.980
Non-white	84 (68.9)	101 (68.7)	185 (68.8)	
Has religious beliefs				
Yes	98 (80.3)	101 (68.7)	199 (74.0)	0.031
No	24 (19.7)	46 (31.3)	70 (26.0)	
Has had sexual intercourse				
Yes	59 (48.4)	77 (52.4)	136 (50.6)	0.511
No	63 (51.6)	70 (47.6)	133 (49.4)	
Lives with parents				
Yes	110 (90.2)	134 (91.2)	244 (90.7)	0.780
No	12 (9.8)	13 (8.8)	25 (9.3)	
Has repeated a grade				
Yes	33 (27.0)	55 (37.4)	88 (32.7)	0.071
No	89 (73.0)	92 (62.6)	181 (67.3)	
Receives Bolsa Família				
Yes	19 (15.7)	32 (22.1)	51 (19.2)	0.189
No	102 (84.3)	113 (77.9)	215 (80.8)	
Income				
< 1 minimum salary	72 (60.0)	91 (62.8)	163 (61.5)	0.646
> 1 minimum salary	48 (40.0)	54 (37.2)	102 (38.5)	
	**Median (95% CI)**	**Median (95% CI)**	**Median (95% CI)**	**p**^b^
Age	16 (16–16)	16 (16–16)	16 (16–16)	0.310

a Chi-square test;

b Mann-Whitney test. n, number of cases; CI, Confidence Interval.

The comparison of sociodemographic characteristics between the female and the male students yielded a significant statistical difference only in terms of the variable “has religious beliefs” (p = 0.037).

A sex-based analysis of the student's responses to questions about their knowledge, attitudes, and preventive practices with respect to HPV and STIs, along with the 95% CI, prevalence ratio, and p results, is shown in Table 2.

**Table 2:** Knowledge, attitudes, and preventive practices of public high school students with regard to HPV and other STIs according to sex.

Characteristics	Female	Male	Total	PR (95% CI)^a^	p^a^	PR (95% CI)^b^	p^a^
Do you know what HPV is?							
Yes	89 (73.0)	53 (36.1)	142 (52.8)	1.29 (1.19–1.40)	<0.001	1.26 (1.16–1.37)	<0.001
No	33 (27.0)	94 (63.9)	127 (47.2)				
Do you know how it is transmitted?							
Yes	85 (69.7)	84 (57.1)	169 (62.8)	1.10 (1.01–1.19)	0.032	1.09 (1.00–1.19)	0.054
No	37 (30.3)	63 (42.9)	100 (37.2)				
Do you know how it is prevented?							
Yes	93 (76.2)	88 (59.9)	181 (67.3)	1.13 (1.04–1.23)	0.003	1.12 (1.03–1.23)	0.007
No	29 (23.8)	59 (40.1)	88 (32.7)				
Have you ever been concerned with HPV?							
Sim	59 (48.4)	47 (32.0)	106 (39.4)	1.11 (1.03–1.19)	0.006	1.10 (1.02–1.19)	0.011
No	63 (51.6)	100 (68.0)	163 (60.6)				
Have you ever sought health care due to concerns about HPV?							
Yes	26 (21.3)	8 (5.4)	34 (12.6)	1.09 (1.04–1.14)	<0.001	1.09 (1.04‒1.14)	<0.001
No	96 (78.7)	139 (94.6)	235 (87.4)				
Do you know what Pap Smear is?							
Yes	79 (64.8)	43 (29.3)	122 (45.4)	1.23 (1.12–1.34)	<0.001	1.24 (1.13–1.36)	<0.001
No	43 (35.2)	104 (70.7)	147 (54.6)				
Do you know what the cervix is?							
Yes	92 (75.4)	65 (44.2)	157 (58.4)	1.25 (1.15–1.35)	<0.001	1.23 (1.13–1.34)	<0.001
No	30 (24.6)	82 (55.8)	112 (41.6)				
Do you know what cancer is?							
Yes	119 (97.5)	138 (93.9)	257 (95.5)	1.04 (1.00–1.08)	0.129	1.03 (0.98–1.08)	0.237
No	3 (2.5)	9 (6.1)	12 (4.5)				
Do you know what cervical cancer is?							
Yes	72 (59.0)	59 (40.1)	131 (48.7)	1.13 (1.05–1.23)	0.002	1.13 (1.04–1.22)	0.004
No	50 (41.0)	88 (59.9)	138 (51.3)				
Do you know what STI is?							
Yes	110 (90.2)	126 (85.7)	236 (87.7)	1.04 (0.97–1.12)	0.260	1.03 (0.96–1.11)	0.337
No	12 (9.8)	21 (14.3)	33 (12.3)				
Do you know how to prevent it?							
Yes	110 (90.2)	122 (83.0)	232 (86.2)	1.07 (0.99–1.14)	0.080	1.05 (0.98–1.13)	0.151
No	12 (9.8)	25 (17.0)	37 (13.8)				
Do you know what a condom is?							
Yes	112 (91.8)	135 (91.8)	247 (91.8)	1.00 (0.94–1.06)	0.992	1.00 (0.94–1.07)	0.951
No	10 (8.2)	12 (8.2)	22 (8.2)				
Do you know someone who has had a sexually transmitted disease?							
Yes	25 (20.5)	25 (17.0)	50 (18.6)	1.02 (0.97–1.07)	0.469	1.01 (0.96–1.07)	0.733
No	97 (79.5)	122 (83.0)	219 (81.4)				

a Crude model;

b Adjusted Model by income, age, ethnicity, grade retention, cohabitation with parents, and religion. PR, Prevalence ratio; CI, Confidence Interval.

The use of the adjusted Poisson regression model with robust variance revealed a difference in the answers of boys and girls. The following questions had statistically different answers when comparing the female and the male adolescents: “Do you know what HPV is?” (PR = 1.26 [1.16‒1.37], p < 0.001); “Do you know how it is prevented?” (PR = 1.12 [1.03‒1.23], p = 0.007); “Have you ever been concerned with HPV?” (PR = 1.10 [1.02‒1.19], p = 0.011); “Have you ever sought health care due to concerns about HPV?” (PR = 1.09 [1.04‒1.14], p < 0.001); “Do you know what a Pap Smear is?” (PR = 1.24 [1.13‒1.36], p < 0.001); “Do you know what the cervix is?” (PR = 1.23 [1.13‒1.34], p < 0.001); “Do you know what cervical cancer is?” (PR = 1.13 [1.04‒1.22], p = 0.004). A statistical difference was also found in the crude model, which also exhibited a difference in relation to the question “Do you know how it is transmitted?” (PR = 1.10 [1.01‒1.19], p = 0.032) (Table 2).

## Discussion

In adolescence, the desire for sexual experience frequently predisposes young people to risky sexual habits due to their immaturity and inexperience. Access to information and health professionals can assist with guidance and the acquisition of healthy habits.^10^

The present study indicates that adolescents know little about STIs, that less than 40% show some concern about HPV, and that only 12.6% have sought health care due to worries about HPV. The male sex was less knowledgeable and concerned than the female sex.

Studies of knowledge about HPV/STI are important guidelines in public health, as they show that gaps in understanding can be barriers to the self-care process and to adherence to vaccination programs. The consequences of HPV are not immediate since cancer precursor lesions and HPV-induced lesions take a while to be detected and treated, with repercussions that include high mortality from these cancers, mainly in developing countries.^11^^,^^12^

The sample was characterized by a majority population of Afro-descendants. This epidemiological profile is similar to that of other studies with parallel topics. The predominance of students of African descent in public schools should be highlighted. Racial inequality among low-income adolescents in Brazil is a factor that may have a negative psychological influence, especially when one feels discriminated against by their ethnicity.^13^ Moreover, the lack of public policies for the dissemination of accessible knowledge among the less favored social class could explain the low level of knowledge of students about their health. This way, greater investments in education and health in this age group, along with religion, may increase interest in sexual health in developing countries such as Brazil.^14^

The assessment of homogeneity between characteristics of male and female adolescents shows that the variable ‘has religious beliefs’ is answered positively more often by the female population. The studied country is predominantly Catholic, and the Church provides information on reproductive and sexual health,^15^ which may be related to the authors’ findings. Religious belief and practice can interfere with knowledge, attitudes, and prevention related to HPV and other STIs, making public health planning interventions difficult by hindering adherence or by offering resistance to the search for health.^16^

An important point in the present study is the difference in knowledge about STIs, including HPV, between the male and the female sexes: the latter know more about the subject than the former. Many factors may be associated with the discrepancy between the sexes. The study by Genz et al. (2017)^17^ reported that girls talked more about sex with their mothers than boys. This factor may have predisposed female adolescents to seek more information regarding STIs (and, consequently, HPV), thus deepening their understanding of the virus and of ways to prevent the disease.^18^

Besides, in Brazil, the implementation of the government's HPV vaccine program in schools, initially was limited to girls and their parents, which may have contributed to the differences in knowledge between the sexes.^19^

In the study by Sousa et al. (2018),^20^ students, particularly those in the 10 to 14 age group, displayed scant knowledge of the HPV vaccine. This population deserves attention as it is going through a period of physical changes, emotional instability, and exposure to new experiences, which makes them more vulnerable and thus subject to contracting HPV and other STIs through risky behavior or unprotected sex.^21^

The results of this study reinforce the need for health promotion actions with adolescents – especially male students ‒ in order to improve their knowledge about HPV and/or STIs beyond the dissemination of such knowledge in schools,^22^ especially among male students. This point needs to be investigated in future studies to determine the real motives for the boys’ low interest.

This shows a clear need to address the male population; even if male adolescents have not been strongly affected by HPV, they may have transmitted the virus and spread it among the female population.^23^ As its stands today, an HPV vaccination campaign is less effective in reaching the male audience.^24^

Cervical cancer has HPV as its main etiological viral factor and other STIs as associated factors, being highly prevalent in Brazil.^25^ However, it is not just women who should be concerned about HPV, because although 70% of men with HPV infection remain asymptomatic, it can result in a spectrum of genitourinary manifestations such as genital warts, penile intraepithelial neoplasia, and even penile carcinomas.^26^

Thus, there is a need to intensify campaigns, which should aim mostly at public school male teenagers. Not only chemical and biological processes, but also intense and unstructured emotions occur in this phase, possibly triggering affective relationships that become the gateway to erroneous, premature, and unprotected sex predisposing adolescents to contract STIs.^17^

One of the limitations of this cross-sectional study is that the present study's sample consisted of adolescents from public schools. This narrow selection of subjects may have biased the variable ‘knowledge and perception’ because it does not necessarily express the reality of other teenagers with the same socioeconomic level or access to education.

Another limiting factor is the use of quantitatively analyzed objective questionnaires, where students were asked whether or not they had knowledge about the subjects in question, but these answers were not confirmed. In this way, the knowledge investigated is referred/perceived. In addition, the questionnaires do not allow the exploration of socioeconomic aspects, such as religious beliefs. nor the prevalence of STI/HPV and vaccination. Another shortcoming is the fact that this study was not designed to assess the real causes of male disinterest in their health. Therefore, other studies are needed.

The highlights of this study conducted in a poor community include an elucidation of the divergences of sexual health knowledge among adolescents and a comparison of boys’ and girls’ knowledge. Such information can be used to underpin sex education, reproductive health programs, and specific health promotion actions. Public health education should be the focus of pediatric and adolescent gynecologists.

## Conclusion

The presentresults show that about half of the adolescents from public schools in the city of São Paulo do not know what HPV is, nor do they know what cervical cancer is. Indeed, a large part never worried about HPV and the vast majority did not seek a health service due to HPV-related issues. In addition, male adolescents not only know less than female adolescents but are also less concerned about health care.

## Ethics approval

The project was approved by the Ethics Committee of the Faculdade de Medicina da USP. The protocol number is 38719314.2.00000068.

## Consent to participate

The participants were informed about the research objectives, and they signed an informed consent form.

## Authors’ contributions

J.M.S.J.: He made substantial contributions to the concept, study design, and definition of intellectual content; he was involved in literature search, data analysis, statistical analysis, manuscript preparation, and manuscript writing; he drafted the article or revised it critically for important intellectual content, and he approved the final version to be published.

H.M.C.O.: She made substantial contributions to the concept, study design, and definition of intellectual content; she was involved in literature search, data analysis, statistical analysis, manuscript preparation, and manuscript writing; she drafted the article or revised it critically for important intellectual content; and she approved the final version to be published.

C.M.L.: She was involved in literature search, data analysis, statistical analysis, manuscript preparation, and manuscript writing; she drafted the article or revised it critically for important intellectual content.

L.T.S.Z.: She was involved in literature search, data analysis, and statistical analysis; and she approved the final version to be published.

E.C.A.V: He was involved in literature search, data analysis, and statistical analysis; and he approved the final version to be published.

J.Z.R.: She was involved in literature search, data analysis, statistical analysis; and she approved the final version to be published.

F.W.S.F.: He was involved in literature search, data analysis, and statistical analysis; and he approved the final version to be published.

M.S.A.: She was involved in literature search, manuscript preparation, and manuscript writing; and she approved the final version to be published.

I.C.E.S.: She made substantial contributions to the concept, study design, and definition of intellectual content; she was involved in literature search, data analysis, statistical analysis, manuscript preparation, and manuscript writing; she drafted the article or revised it critically for important intellectual content; and she approved the final version to be published.

E.C.B: He was involved in literature search, data analysis, and statistical analysis; and he approved the final version to be published.

## Funding

This study was supported by FAPESP (#2016/16847-0 and #2017/07276-2), CNPq (#301725/2017-9), and CAPES (Brasília, BR).

## Conflicts of interest

The authors declare no conflicts of interest.

## Appendix Supplementary materials

Image, application 1
