# Supplementary material for: Adolescents’ knowledge of HPV and sexually transmitted infections at public high schools in São Paulo: A cross-sectional study
Source: Clinics (Sao Paulo). 2022 Nov 17;77:100138. doi: 10.1016/j.clinsp.2022.100138 (PMC9678670; doi:10.1016/j.clinsp.2022.100138)
Supplement: Supplementary file 1 [file mmc1.docx]

**CLINICS-D-22-00444 – Supplementary Material**

**Supplementary Material** Questionnaire about the knowledge, attitudes, and preventive practices with regard to STIs, including HPV.

| 1) Do you know what HPV (human papilloma virus) is? |
| --- |
| ( ) I don’t know ( ) I’ve heard of ( ) I know partially ( ) I know a little ( ) I know |
| 2) Do you know how it is transmitted? |
| ( ) I don’t know ( ) I’ve heard of ( ) I know partially ( ) I know a little ( ) I know |
| 3) Do you know how it is prevented? |
| ( ) I don’t know ( ) I’ve heard of ( ) I know partially ( ) I know a little ( ) I know |
| 4) Have you ever been concerned with HPV? |
| ( ) never ( ) sometimes ( ) often ( ) I’m aware ( ) I worried a lot |
| 5) Have you ever sought health care due to concerns about HPV? |
| ( ) never ( ) sometimes ( ) often ( ) I’m aware ( ) I worried a lot |
| 6) Do you know what “Pap Smear” or “Pap Test” is? |
| ( ) I don’t know ( ) I’ve heard of ( ) I know partially ( ) I know a little ( ) I know |
| 7) Do you know what the cervix is? |
| ( ) I don’t know ( ) j I’ve heard of ( ) I know partially ( ) I know a little ( ) I know |
| 8) Do you know what cancer is? |
| ( ) I don’t know ( ) I’ve heard of ( ) I know partially ( ) I know a little ( ) I know |
| 9) Do you know what cervical cancer is? |
| ( ) I don’t know ( ) I’ve heard of ( ) I know partially ( ) I know a little ( ) I know |
| 10) Do you know what sexually transmitted disease is? |
| ( ) I don’t know ( ) I’ve heard of ( ) I know partially ( ) I know a little ( ) I know |
| 11) Do you know how to prevent it? |
| ( ) I don’t know ( ) I’ve heard of ( ) I know partially ( ) I know a little ( ) I know |
| 12) Do you know what a condom is? |
| ( ) I don’t know ( ) I’ve heard of ( ) I know partially ( ) I know a little ( ) I know |
| 13) Do you know someone who has had a sexually transmitted disease? |
| ( ) I don’t know ( ) I’ve heard of ( ) I know partially ( ) I know a little ( ) I know |
